# Verb fluency task in individuals with mild cognitive impairment and dementia: a scoping review

**DOI:** 10.1002/alz70857_101887

**Published:** 2025-12-24

**Authors:** Emily Viega Alves, Fernanda Mambrini Só e Silva, Victória Tizeli Souza, Bárbara Costa Beber, Raphael Machado Castilhos

**Affiliations:** ^1^ Federal University of Rio Grande do Sul, Porto Alegre, Rio Grande do Sul, Brazil; ^2^ Universidade Federal de Ciências da Saúde de Porto Alegre (UFCSPA), Porto Alegre, Rio Grande do Sul, Brazil; ^3^ Universidade Federal do Rio Grande do Sul, Porto Alegre, Rio Grande do Sul, Brazil

## Abstract

**Background:**

The verb fluency task is considered more challenging than other verbal fluencies and may aid in early diagnosis of Mild Cognitive Impairment (MCI) and dementia. The aim of this work was to carry out a scoping review of studies that investigated the assessment of the verb fluency task in individuals with MCI and dementia.

**Method:**

This is a scoping review conducted according to the PRISMA‐ScR. Papers included needed to measure verb fluency task in individuals with MCI or dementia, with no restrictions on publication period or language. Studies that did not assess verb fluency or focused on other types of verbal fluency in individuals without MCI or dementia were excluded. The databases used for the search were PubMed, LILACS, Scielo, Embase and Scopus. The variables sought were related to the methodology used for assessment, specific patterns produced by the population and the ability of the task to distinguish MCI from dementia.

**Result:**

Two reviewers initially screened 977 publications, excluding 269 duplicates. The remaining 708 were assessed by title and abstract, resulting in 37 studies selected for full reading, with 16 included in the scoping review. The majority of publications (93.7%) reported cross‐sectional studies. 87.5% used the methodology of analyzing correct verbs, while 25% also used clustering and switching assessment. Error analysis was only conducted in 2 studies. In general (75%), the studies considered a task duration of 1 minute. Individuals with dementia showed significantly greater impairments in verb fluency compared to those with MCI, who also demonstrated reduced performance relative to healthy individuals. The deficits in this task highlight underlying cognitive changes, such as in working memory and executive functions. The temporal lobe was the main identified as dysfunctional in individuals who performed worse.

**Conclusion:**

Despite having diverse aims, the works were similar in terms of study design and task assessment methodology. The main result of this scoping review is that the verb fluency task is a sensitive tool for distinguishing individuals with MCI from those with dementia and can highlight underlying cognitive deficits. Future studies should prioritize qualitative analyses, as well as longitudinal findings.

